# Promoter analysis of intestinal genes induced during iron-deprivation reveals enrichment of conserved SP1-like binding sites

**DOI:** 10.1186/1471-2164-8-420

**Published:** 2007-11-15

**Authors:** James F Collins, Zihua Hu

**Affiliations:** 1 Department of Exercise and Nutrition Sciences, University at Buffalo, the State University of New York, Buffalo, NY, USA; 2 Center for Computational Research, New York State Center of Excellence in Bioinformatics & Life Sciences, and the Department of Biostatistics, University at Buffalo, the State University of New York, Buffalo, NY, USA

## Abstract

**Background:**

Iron-deficiency leads to the induction of genes related to intestinal iron absorption and homeostasis. By analyzing a large GeneChip^® ^dataset from the rat intestine, we identified a large cluster of 228 genes that was induced by iron-deprivation. Only 2 of these genes contained 3' iron-response elements, suggesting that other regulation including transcriptional may be involved. We therefore utilized computational methods to test the hypothesis that some of the genes within this large up-regulated cluster are co-ordinately regulated by common transcriptional mechanisms. We thus identified promoters from the up-regulated gene cluster from rat, mouse and human, and performed enrichment analyses with the Clover program and the TRANSFAC database.

**Results:**

Surprisingly, we found a strong statistical enrichment for SP1 binding sites in our experimental promoters as compared to background sequences. As the TRANSFAC database cannot distinguish among SP/KLF family members, many of which bind similar GC-rich DNA sequences, we surmise that SP1 or an SP1-like factor could be involved in this response. In fact, we detected induction of SP6/KLF14 in the GeneChip^® ^studies, and confirmed it by real-time PCR. Additional computational analyses suggested that an SP1-like factor may function synergistically with a FOX TF to regulate a subset of these genes. Furthermore, analysis of promoter sequences identified many genes with multiple, conserved SP1 and FOX binding sites, the relative location of which within orthologous promoters was highly conserved.

**Conclusion:**

SP1 or a closely related factor may play a primary role in the genetic response to iron-deficiency in the mammalian intestine.

## Background

Iron is a critical element required for normal homeostasis. This fact is best exemplified by the role of iron in diverse physiological processes such as DNA synthesis, respiration and oxygen transport. Body iron levels must be precisely maintained within certain limits as iron-deficiency and iron-overload result in perturbations of normal metabolism. To achieve appropriate levels of cellular iron and to avoid iron-loading, several mechanisms have evolved; these include the control of iron transport, storage and recycling by regulatory proteins. A key step in controlling overall body iron levels is absorption of dietary iron in the duodenum. Iron absorption is a highly regulated process, as mammals have no controlled means to excrete excess iron. Recently, several key proteins involved in intestinal iron transport have been identified. These include the brush-border membrane associated proteins duodenal cytochrome b (Cybrd1; a ferrireductase), and divalent metal transporter 1 (Dmt1 or Slc11a2), which together allow dietary iron to be reduced and transported into enterocytes. The combined activity of hephaestin (Hp; a ferroxidase) and ferroportin (Fpn; the basolateral iron exporter) subsequently allows oxidized iron to exit the cell and bind to transferrin for distribution throughout the body. Intestinal iron transport is in part regulated by the liver-derived, antimicrobial peptide, hepcidin. Under conditions of iron excess, elevated hepcidin acts to remove Fpn from the basolateral surface of enterocytes effectively creating a mucosal block to iron [1].

Iron-deprivation increases expression of genes involved in intestinal iron-transport in laboratory rodents [2,3] and in humans [4]. Some of these induced genes are regulated post-transcriptionally via the iron regulatory protein (IRP)/iron-response element (IRE) system. These genes include ferritin, transferrin receptor (Tfr) and possibly Dmt1 [5] and Fpn [6]. Despite the large body of work describing these regulatory events, many transcripts encoding proteins involved in iron homeostasis do not have IREs and it is thus very likely that transcriptional regulation may also be important [7]. Despite the wealth of knowledge regarding transcriptional regulation of gene expression in response to iron-deprivation in lower species such as yeast [8], no such regulatory networks have been identified to date in mammals.

Our previous studies were the first that utilized a genome wide approach to identify genes regulated during iron-deficiency in the mammalian duodenum [2,3]. We identified a large number of differentially expressed genes (DEGs), many of which had never been described to be regulated by iron or body iron status. In the current manuscript, we utilized computational and bioinformatics approaches to identify regulatory mechanisms that may mediate the genetic response to iron deprivation. We thus first used clustering algorithms to group genes from our previous GeneChip^® ^studies to identify groups of co-regulated genes. Of particular interest was a group of 228 probe sets (representing ~168 genes) that were up-regulated across several different stages of postnatal development. Interestingly, of these genes, only the *Dmt1 *and *Tfr *genes contained 3' IREs, suggesting that analysis of transcriptional regulatory regions could be a fruitful approach to identify common regulatory mechanisms. Our subsequent analyses found that SP1-like binding sites were statistically enriched in promoters from these genes in rat and were conserved across 3 mammalian species. Additional studies suggested that SP and FOX transcription factors may work synergistically to regulate some genes during iron-deprivation; in fact, we found conserved FOX binding sites in many genes with conserved SP1 binding sites. We thus hypothesize that SP and FOX family transcription factors are involved in regulating expression of a subset of intestinal genes induced during iron-deficiency. We also found strong induction (~30-fold) of specificity factor 6 (SP6) by qRT-PCR, suggesting that it may be the SP family member that mediates the genetic response to iron-deprivation. Overall, these data suggest the existence of a transcriptional regulatory network(s) which responds to iron intake levels or other physiological signals associated with iron-deficiency in the mammalian intestine.

## Results

**Figure 1:**
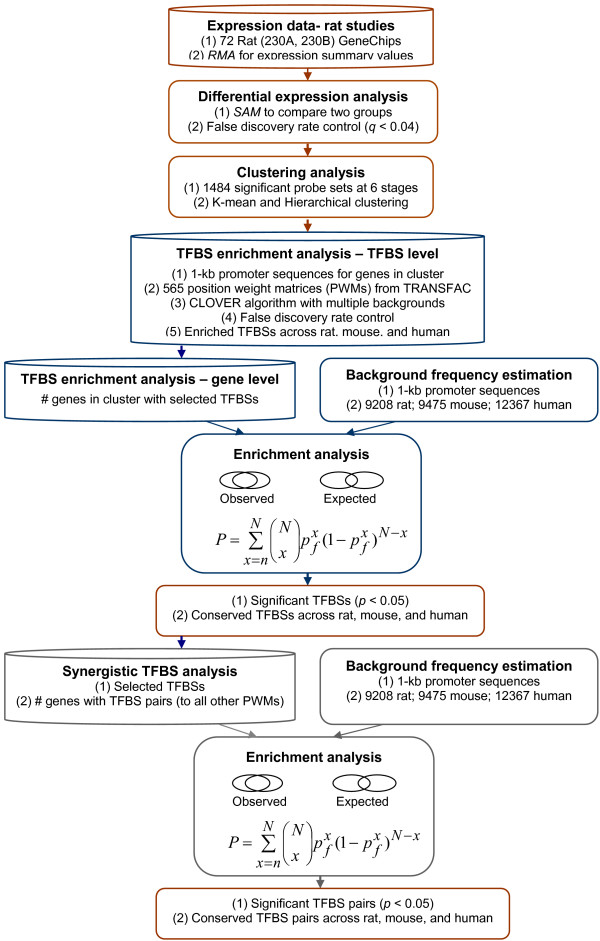
**Schematic of Overall Data Analysis Approach**. Gene chip data were analyzed using the RMA probe set algorithm. We then used the Statistical Analysis of Microarray (SAM) approach with control of false discovery rate to identify 1484 differentially expressed genes across our experimental groups. Clustering analysis was then performed; promoters of genes from clusters of interest were then identified and TFBS enrichment analysis was performed. This was accomplished with PWMs from Transfac and the CLOVER algorithm. For estimation of the background frequency of TFBSs, we compared our experimental data to thousands of random promoter sequences. We also performed enrichment analysis at the gene level, which told us if certain TFBSs were present in more of our experimental promoters than in random samplings of background promoters. These analyses led to the identification of enriched TFBSs that were conserved in rat, mouse and human. The final analysis allowed us to predict synergistic interactions between TFs by estimating the frequency of the presence of TFBS combinations in our experimental promoters as compared to random, background promoter sequences.

Our overall data analysis approach is summarized in Figure 1. Briefly, computational analysis of the gene chip data allowed us to identify 1484 differentially expressed genes across our experimental groups. Clustering analysis led to the identification of interesting gene clusters that could represent co-regulated genes. To determine potential regulatory mechanisms that lead to the upregulation of some genes during iron-deficiency, we performed TFBS enrichment analysis of promoters from our experimental genes across 3 mammalian species. For background frequency estimation, we used thousands of random promoter sequences from the same species. The final analysis allowed us to predict potential synergistic TF interactions that may be involved in regulating some of our experimental genes.

### Clustering Analysis

**Figure 2:**
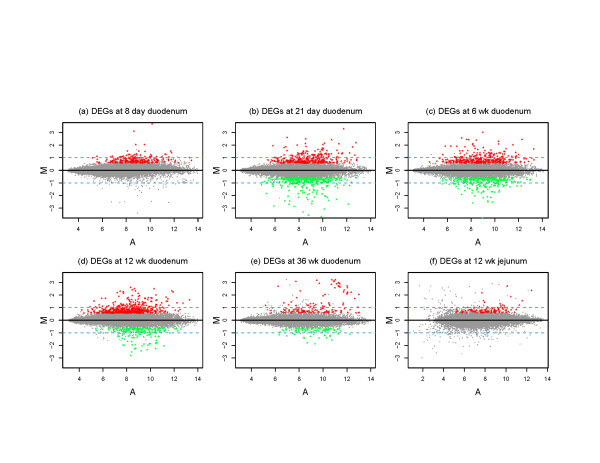
**Scatter Plots of GeneChip Data**. Each panel represents GeneChip data from one experimental group using *M *(log_2 _(average intensity of iron-deficient group/average intensity of control group)) vs. *A *((log_2 _(average intensity of iron-deficient group) + log_2 _(average intensity of control group))/2)) plot. Differentially expressed genes (FDR < 0.04 and fold change > 1.5) are shown in color, including down-regulated genes (in green) and up-regulated genes (in red). Dashed lines indicate 2-fold change

1484 differentially expressed genes were identified across our experimental groups, including some genes that were up- or down-regulated in all groups studied and some genes that were regulated in as few as one experimental group. These genes are depicted visually in scatter plots shown in Figure 2, where up-regulated genes are seen as red dots and down-regulated genes are seen as green dots. From this representation, it is apparent that in suckling rats and in adult rat jejunum, there were no down-regulated genes that met the statistical cut-offs.

**Figure 3:**
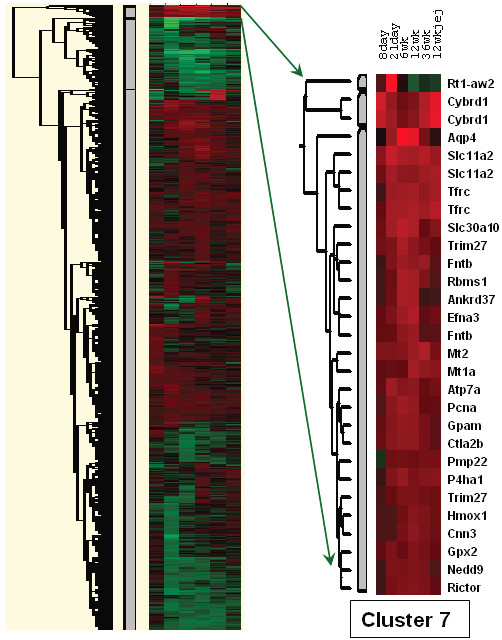
**Hierarchical Clustering**. On the left is shown a heat map that resulted from the clustering analysis. Each of the 6 columns represents one of the 6 experimental groups. Red color represents up-regulated genes and green color represents down-regulated genes; the brighter the red or green, the greater the magnitude of change. Black color indicates genes that were not differentially expressed. Cluster 7 is shown on the right along with corresponding gene symbols. The different experimental groups are indicated above.

Two different approaches were utilized to identify groups of potentially co-regulated genes, hierarchical and K-mean clustering. K-mean clustering led to three subsets of genes; one group was predominantly up-regulated, one group was mostly down-regulated and one group showed a mixed response (data not shown). Of particular interest was a group of 228 up-regulated probe sets (Additional File 1), as this group contained several genes known to be involved in the genetic response to iron-deprivation, including *Dmt1*, *Cybrd1 *and *Tfr*. Hierarchical clustering led to the identification of many groups of genes (Figure 3); one cluster, which we called cluster 7, contained 29 probe sets, representing 25 up-regulated genes. This cluster was contained within the 228 cluster and it also contained several genes involved in iron homeostasis (Table 1). Moreover, gene ontology (GO) analysis of both the 228 up-regulated cluster and cluster 7 revealed statistical enrichment for gene functions related to iron and metal ion homeostasis (Table 2), supporting the validity of our analyses.

**Table 1:** Up-Regulated Gene Cluster 7

**Probe Set ID**	**Symbol**	**Gene Name; Aliases**
1373685_at	Ankrd37	Ankyrin repeat domain 37; Similar to Lrp2bp-pending protein
1372190_at	Aqp4	Aquaporin 4
1392536_at	Atp7a	ATPase, Cu^++ ^transporting, alpha polypeptide
1387856_at	Cnn3	Calponin 3, acidic
1389659_at	Ctla2b	Cytotoxic T lymphocyte-associated protein 2 beta precursor
1376344_at	Cybrd1	Cytochrome b reductase 1-similar
1377369_at	Cybrd1	Cytochrome b reductase 1 (predicted)
1390763_at	Efna3	Ephrin A3
1370829_at	Fntb	Farnesyltransferase, CAAX box, beta
1372452_at	Gpam	Glycerol-3-phosphate acyltransferase, mitochondrial
1374070_at	Gpx2	Glutathione peroxidase 2
1370080_at	Hmox1	Heme oxygenase (decycling) 1
1371237_a_at	Mt1a	Metallothionein
1388271_at	Mt2	Metallothionein-2
1374001_at	NanogPc	Retrotransposon NANOGPC gene; same chromosomal region
1374650_at	Nedd9	Neural precursor cell expressed, developmentally down-regulated gene 9 (predicted)
1370954_at	P4ha1	Procollagen-proline, 2-oxoglutarate 4-dioxygenase (proline 4-hydroxylase), α1 polypeptide
1367671_at	Pcna	Proliferating cell nuclear antigen
1370247_a_at	Pmp22	Peripheral myelin protein 22
1373488_at	Rbms1	RNA binding motif, single stranded interacting protein 1 isoform c-similar
1372197_at	Rictor	Rapamycin-insensitive companion of mTOR; Pianissimo-similar
1370428_x_at	RT1-Aw2	RT1 class Ib, locus Aw2
1367877_at	Slc11a2	Solute carrier family 11 (proton-coupled divalent metal ion transporters), member 2
1388059_a_at	Slc11a2	Solute carrier family 11 (proton-coupled divalent metal ion transporters), member 2
1383913_at	Slc30A10	Zinc transporter 8; Znt8
1371113_a_at	Tfrc	Transferrin receptor
1388750_at	Tfrc	Transferrin receptor
1377234_at	Trim27	Tripartite motif protein 27 (predicted)
1371737_at	Trim27; Rfp	Tripartite motif protein 27; Ret finger protein

**Table 2:** Gene Ontology Analysis

**Gene Ontology Term-Cluster 7**	***p *Value**
Cadmium ion binding	0.002
Iron ion transporter activity	0.006
Iron ion binding	0.01
Transition metal ion binding	0.02
Iron ion transport	0.03
Transition metal ion transporter activity	0.03
Transition metal ion homeostasis	0.04
Cell proliferation	0.04
Ion binding	0.05
Metal ion binding	0.05

**Gene Ontology Term-228 Cluster**	***p *Value**

Oxidoreductase activity	0.000040
Transition metal ion binding	0.0053
Ion binding	0.0054
Metal ion binding	0.0054
Catalytic activity	0.0074
Cadmium ion binding	0.0.15
Transition metal ion transporter activity	0.015
Dioxygenase activity	0.018
N-acyltransferase activity	0.026
Iron ion binding	0.033
Iron ion transporter activity	0.037
Vitamin binding	0.045
Acyltransferase activity	0.046

### Iron-Response Element (IRE) Analysis

Transcripts representing genes in the 228 up-regulated cluster were analyzed for the presence of 3' IREs, as IREs in this part of the transcript could lead to message stabilization during iron-deficiency (and thus lead to increases in steady state mRNA levels). We found 3' IREs in only the *Dmt1 *and *Tfr *transcripts (data not shown).

### Single Transcription Factor Binding Site (TFBS) Analysis

5' proximal promoters for available genes in the 228 up-regulated cluster were subjected to computational analysis to search for over-represented TFBSs. The analysis results revealed a strong statistical enrichment for SP1 binding sites in our experimental promoters across 3 mammalian species, as compared to thousands of random promoter sequences from the same 3 species (Table 3). Four SP1 position weight matrices (PWMs) were identified and all had very high raw scores, which mean that the identified binding sites are very similar to consensus binding sites [9]. While other PWMs also showed significance in individual species (not shown), none except SP1 was significant across rat, mouse and human promoters. It should be noted that the TRANSFAC program has PWMs for  SP1, SP3 and 2 KLF family members; however, there are over 20 SP/KLF  proteins and many of them have high amino acid sequence homology in the DNA binding regions (i.e. the zinc fingers) and they thus bind similar GC-rich regions [10]. Utilizing a complimentary approach, it was further demonstrated that SP1-like binding sites were also enriched at the gene level, in that our group of experimental promoters had a higher percentage of genes with SP1-like sites than random samplings of background promoter sequences (Table 4).

**Table 3:** Clover *Cis*-Element Over-Representation: Common Enriched TFBSs Among Species

**TFBSs (PWM)**	**Rat**		**Mouse**		**Human**	
	Raw Score	*p*	Raw Score	*p*	Raw Score	*p*

V$SP1_Q6_01	177	0.017	235	< 0.001	217	< 0.001
V$SP1_Q2_01	192	0.031	248	< 0.001	240	< 0.001
V$SP1_Q4_01	188	0.038	256	< 0.001	231	< 0.001
V$SP1_Q6	175	0.043	242	< 0.001	235	< 0.001
						
**Background Sequences:**	9,201 Rat Promoter Sequences (9,215,186 bp; 51.4% GC)					
	9,475 Mouse Promoter Sequences (9,483,474 bp; 49.2% GC)					
	12,367 Human Promoter Sequences (12,379,367 bp; 53.5% GC)					
						
**Experimental Promoters:**	133 Rat Promoters (51.6% GC)					
	144 Mouse Promoters (51.4% GC)					
	159 Human Promoters (54.7% GC)					

**Table 4:** Single TFBS Enrichment: Gene Level

**TFBSs (PWM)**	**Rat *p *value**	**Mouse *p *value**	**Human *p *value**
**V$SP1_Q2_01**	0.004	< 0.001	0.008
**V$SP1_Q4_01**	0.029	< 0.001	< 0.001
**V$SP1_Q6**	0.003	< 0.001	0.002
**V$SP1_Q6_01**	0.06	< 0.001	0.032

Furthermore, analysis of promoter regions of genes within the 228 up-regulated cluster revealed many highly conserved, putative SP1 binding sites (Additional File 2); in many cases, the nucleotide sequence was identical and the spacing between predicted sites was very similar across mammalian species. Our observations thus indicate that SP1 or a related factor may be involved in regulating a subset of genes in the 228 up-regulated cluster during iron-deficiency. In fact, a literature search of genes in this cluster with predicted SP1 sites that were conserved across three mammalian species revealed that previous investigation of many of these genes implicated SP1 in their transcriptional regulation (Table 5). GO analysis of these genes containing conserved, putative SP1 binding sites also revealed enrichment for gene functions related to iron and metal ion homeostasis (Table 6).

**Table 5:** Genes From 228 Cluster With Conserved Sp1 Sites

**Gene Name; Aliases**	**Symbol**	**Evidence Related to Sp-Like Factors**	**Reference**
5'-nucleotidase, cytosolic III	Nt5c3	Unknown	
Activating transcription factor 3	Atf3	Induced by deferroxamine treatment; Sp1 reg. unknown	*Oncogene *26(2):284-9, 2007.
Ankyrin repeat domain 37	Ankrd37	Unknown	
Aquaporin 4	Aqp4	Promoter has consensus Sp1 sites	*Genomics *50(3):373-7, 1998.
ATPase, Cu^++ ^transporting, alpha polypeptide (Menkes syndrome)	Atp7a	Unknown	
ATP-binding cassette, sub-family G (WHITE), member 2	Abcg2	Several consensus Sp1 sites within minimal functional promoter	*Biochim. Biophys. Acta*. 1520(3):234-41, 2001.
Axin 2 (conductin, axil)	Axin2	Unknown	
BCL2-like 11 (apoptosis facilitator)	Bcl2l11	Bcl2l11 inc. with apoptosis; enrichment for Sp1/Sp3 sites	*Biochim. Biophys. Acta*1693:167–176, 2004.
Tubulin polymerization-promoting protein; Brain-specific protein p25 alpha	Tppp	Unknown	
Calponin 3, acidic	Cnn3	Unknown	
Chemokine (C-C motif) ligand 20	Ccl20	Reg. by Sp1; MIP-3alpha	*J. Biol. Chem*. 278(2):875-84, 2003.
Cytochrome B Reductase 1	Cybrd1	Unknown	
Cytochrome P450, family 51, subfamily A, polypeptide 1	Cyp51a1	Sp1 maximizes sterol regulation of promoter	*Molecular Endocrinology *16 (8): 1853–1863, 2002.
DNA-damage-inducible transcript 4	Ddit4	Sp1 regulation; hypoxic induction	*Pharm. Res*. 21(5):736-41, 2004.
Early growth response 1	Egr1	Binds and inhibits Sp1	*Am. J. Physiol. Renal Physiol. *284(6):F1216-25, 2003.
	Egr1	Promoter has consensus Sp1 sites	*Oncogene *6(5):867-71, 1991.
	Egr1	Sp1 activates Egr1 gene expression	*J. Biol. Chem*. 268(23):16949-57, 1993.
	Egr1	Sp1 and Egr1 bind similar GC rich	*Biochem. Biophys. Res. Commun*. 194(3):1475-82, 1993.
Ephrin A3	Efna3	Unknown	
ERO1-like (S. cerevisiae); Endoplasmic oxidoreductin-1-like protein	Ero1l	Reg. by hypoxia and Df; Sp1 regulation unknown	*Eur. J. Biochem*. 270(10):2228-35, 2003.
Farnesyltransferase, CAAX box, beta	Fntb	Unknown	
Gap junction protein, beta 2, 26 kDa; Connexin 26	Gjb2	Human gene reg. by Sp1/Sp3	*Biochim. Biophys. Acta*. 1443(1–2):169-81, 1998.
Glutathione peroxidase 2 (gastrointestinal)	Gpx2	Contains Sp1 elements	*Gene *248(1–2):109-16, 2000.
Heme oxygenase 1	Hmox1	Functional Sp1-like site present in PI3 kinase responsive region	*Free Radic. Biol. Med*. 15;41(2):247-61, 2006.
Jumonji domain containing 1A	Jmjd1a	Unknown	
Metallothionein 1A	Mt1a	Sp1 interacts with chicken Mt promoter	*Comp. Biochem. Physiol. Biochem. Mol. Biol*. 116(1):75–86, 1997.
	Mt1a	Sp1 interacts with mouse Mt1 promoter	*Biochem. J. *323 (Pt 1):79–85, 1997.
	Mt1a	Sp1 interacts with metal response element in Mt promoter	*FEBS Lett*. 416(3):254-8, 1997.
	Mt1a	Sp1 interacts with Mt1 promoter	*J. Neurosci*. 20(14):5200-7, 2000.
	Mt1a	Sp1 interacts with Mt1 promoter	*Nucleic Acids Res*. 31(23):6710-21, 2003.
Peripheral myelin protein 22	Pmp22	Unknown	
Phosphoglycerate kinase 1	Pgk1	Sp1 interacts with promoter; *In vivo *footprinting	Genes Dev. 4(8):1277-87, 1990.
Procollagen-proline, 2-oxoglutarate 4-dioxygenase (proline 4-hydroxylase), alpha 1 polypeptide	P4ha1	Unknown	
Proliferating cell nuclear antigen	Pcna	3 conserved Sp1 sites present in murine promoter	*DNA Seq*. 2(3):181-91, 1991.
Ras homolog gene family, member B	RhoB	Contains Sp1 sites	*J. Biol. Chem*. 272(49):30637-44, 1997.
	RhoB	Promoter contains consensus Sp1 sites	*Biochem. Biophys. Res. Commun*. 226(3):688-94, 1996.
Rictor; FYN binding protein (FYB-120/130)	Rictor	Unknown	
Sepiapterin reductase (7,8-dihydrobiopterin:NADP+ oxidoreductase)	Spr	Promoter contains Sp1 sites	*Biochem. Biophys. Res. Commun*. 251(2):597–602, 1998.
Solute carrier family 11 (proton-coupled divalent metal ion transporters), member 2	Slc11a2	Promoter contains Sp1 sites	*Blood Cells Mol. Dis*. 24(2):199–215, 1998.
Solute carrier family 6 (neurotransmitter transporter, taurine), member 6	Slc6a6	Promoter cloning paper	*Adv. Exp. Med. Biol*. 483:97–108, 2000.
Syndecan 1	Sdc1	Promoter reg. by Sp1 factor(s)	*J. Biol. Chem*. 271(21):12532-41, 1996.
TGFB-induced factor (TALE family homeobox)	Tgif	Unknown	
TIMP metallopeptidase inhibitor 1	Timp1	Sp1 basal and hypoxic regulation	*J. Cell. Biochem*. 91(6):1260-8, 2004.
	Timp1	Sp1 invovled in induction by TGF-beta	*J. Cell. Physiol*. 203(2):345-52, 2005.
	Timp1	Sp1 invovled in induction by TGF-beta	*Mol. Cancer Res. *4(3):209-20, 2006.
	Timp1	Sp1 binding increased by leptin treatment	*Mol. Endocrinol*. 20(12):3376-88, 2006.
Transferrin receptor (p90, CD71)	Tfrc	Contains a GC rich region	*Oncogene *21(52):7933-44, 2002.
Tripartitie motif protein 27	Trim27	Sp1 involved in promoter activity	*Biochem. Biophys. Res. Commun*. 261(2):381-4, 1999.
Tubulin-specific chaperone c	Tbcc	Unknown	

**Table 6:** Gene Ontology Analysis of Genes With Conserved Sp1 Sites

**Gene Ontology Term**	***p *Value**	**Genes**
Transition metal ion transporter activity	0.0013	Slc11A2, Tfrc, Atp7a
Transition metal ion binding	0.0021	Slc11A2, Fntb, Mt1a, Hmox1, P4ha1, Egr1, Atp7a, Jmjd1a
Transition metal ion homeostasis	0.0026	Mt1a, Tfrc, Atp7a
Cadmium ion binding	0.0044	Slc11A2, Mt1a
Transition metal ion transport	0.0051	Slc11A2, Tfrc, Atp7a
Copper ion binding	0.0059	Slc11A2, Mt1a, Atp7a
Iron ion transporter activity	0.011	Slc11A2, Tfrc
Copper ion transporter activity	0.019	Slc11A2, Atp7a
Cation binding	0.036	Slc11A2, Fntb, Mt1a, Hmox1, P4ha1, Egr1, Atp7a, Jmjd1a
Di-, tri-valent inorganic cation homeostasis	0.039	Mt1a, Tfrc, Atp7a
Di-, tri-valent inorganic cation transport	0.042	Slc11A2, Tfrc, Atp7a
Metal ion homeostasis	0.044	Mt1a, Tfrc, Atp7a
Zinc ion binding	0.047	Slc11A2, Fntb, Mt1a, Egr1, Jmjd1a
Iron ion binding	0.047	Slc11A2, Hmox1, P4ha1
Cell differentiation	0.049	Axin2, RhoB, Pmp22, Egr1, Efna3, Timp1
Iron ion transport	0.05	Slc11A2, Tfrc

### qRT-PCR Analysis of SP6 mRNA Expression

**Figure 4:**
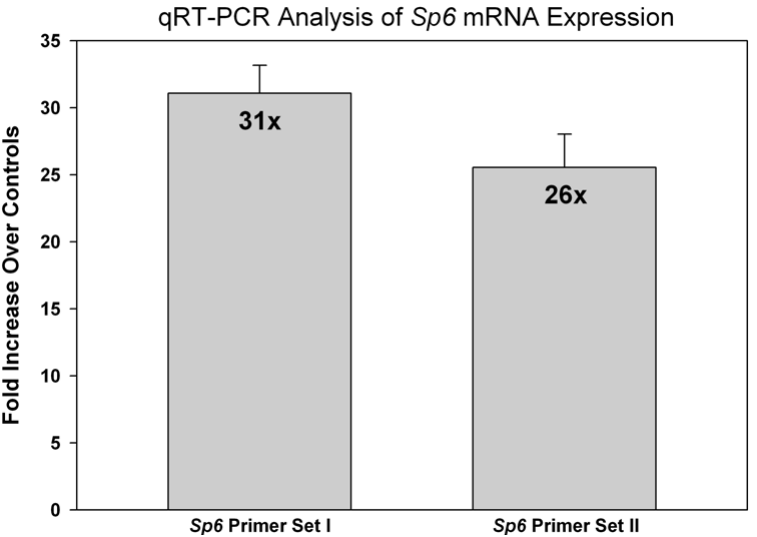
**qRT-PCR Analysis for *SP6 *mRNA Expression**. SYBR Green quantitative RT-PCR was utilized to analyze *SP6 *expression levels in control and iron-deficient rat groups. Fold induction is shown. n = 4 for primer set I and n = 2 for primer set II; *p *< 0.001 between control and iron-deficients for *SP6 *primer set I and *p *= 0.01 for primer set 2. The fold changes detected with primer set I versus primer set II are not different (*p *= 0.78).

The GeneChip^®^ studies revealed induction of *SP6*, but not other Sp/KLF family members; therefore, we designed an experimental strategy to confirm this result. Two different, specific Sp6 primer sets were utilized for real-time PCR analyses, which demonstrated ~30-fold induction of *SP6 *mRNA expression in the iron-deficient rat duodenum (Figure 4). We performed standard curves with each primer set, which showed that they were quantitative over a range of template dilutions (not shown). Furthermore, in some experiments we treated RNA samples with DNase 1 to remove genomic DNA and found that amplification parameters and fold changes were very similar as to when we did not remove the genomic DNA. Melt curves demonstrated that single products were amplified by each primer set.

### Synergistic Transcription Factor Binding Site Analysis

Three SP1 PWMs, V$SP1_Q2_01, V$SP1_Q4_01 and V$SP1_Q6, were significant in the previous analyses at the binding site and gene levels, so we utilized these matrices to search for other TFs that may work in conjunction with an SP1-like factor to regulate intestinal genes during iron-deficiency. All three SP1 PWMs gave similar results in that several TFBSs were identified that were within the statistical parameters we established, including FOX (2 individual PWMs), HNF3, KROX, MZF1 and ROAZ (Table 7). Based upon these findings, we propose that SP1 or an SP1-like factor may work synergistically with one or more of these other TFs to mediate the genetic response to iron-deprivation. Interestingly, we noted that both FOX PWMs and HNF3 binding sites were very similar, and that KROX and MZF1 were highly similar to the predicted SP1 sites. We additionally noted that very few genes in the 228 up-regulated cluster contained ROAZ sites, despite the fact that this cluster contained a strong statistical enrichment for ROAZ and SP1 binding sites. It therefore was apparent that FOX was our strongest candidate for synergistic action with SP1. We were subsequently able to identify several genes in the up-regulated cluster with conserved SP1 and FOX binding sites (Additional File 2).

**Table 7:** Synergistic TFBS Analysis

**Sp1_Q4_01**	Rat *p *value	Mouse *p *value	Human *p *value
**FOX_Q2**	0.002	0.027	0.001
**FOXP3_Q4**	0.004	0.015	0.001
**HNF3_Q6**	0.042	0.013	< 0.001
**KROX_Q6**	0.007	0.001	0.001
**MZF1_02**	0.025	0.017	0.012
**ROAZ_01**	0.005	0.008	0.007

**Sp1_Q2_01**	Rat *p *value	Mouse *p *value	Human *p *value

**FOX_Q2**	0.001	0.025	0.004
**FOXP3_Q4**	0.002	0.022	0.004
**HNF3_Q6**	0.029	0.034	0.001
**KROX_Q6**	0.004	0.001	0.004
**MZF1_02**	0.023	0.041	0.026
**ROAZ_01**	0.003	0.021	0.017

**Sp1_Q6**	Rat *p *value	Mouse *p *value	Human *p *value

**FOX_Q2**	0.006	0.033	0.006
**FOXP3_Q4**	0.004	0.017	0.002
**HNF3_Q6**	0.049	0.007	< 0.001
**KROX_Q6**	0.012	< 0.001	< 0.001
**MZF1_02**	0.045	0.010	0.008
**ROAZ_01**	0.004	0.004	0.009

## Discussion

A well-characterized finding during iron-deficiency in mammals is that genes involved in iron-transport or iron-homeostasis are up-regulated. In some cases, increased gene expression may be mediated post-transcriptionally (via the IRE/IRP system), but transcriptional mechanisms are also likely important [7]. This latter supposition is supported by the identification of iron and metal-responsive transcription factors from a number of lower species that mediate the cellular response to increased or decreased metal ion levels [8,11].

Analysis of a novel GeneChip^® ^dataset resulted in the identification of 1484 DEGs from the duodenum of iron-deficient rats at several postnatal ages, providing us with a unique opportunity to identify groups of induced genes that were potentially coordinately regulated and identify potential mechanisms that could be responsible for this common regulation. Only 2 genes in our up-regulated cluster contained IREs that could potentially mediate increases in steady state mRNA levels. It thus emerged that other levels of regulation (i.e. most likely transcriptional) were likely responsible for the alterations in the expression of this group of genes. We therefore focused on promoter analysis to identify potential candidate regulatory mechanisms.

Gene promoters were identified by a combination of approaches, including manual analysis of probe set IDs from the 228 probe sets in the large up-regulated cluster and BLAST searches that allowed us to analyse alternative GenBank accession numbers for the corresponding cDNAs, which we could link to a promoter sequence in the Cold Spring Harbor Database. Background sequences were assembled as described in the *Methods *section. Transcription factor binding site (TFBS) analysis utilized the Clover program [9] and the TRANSFAC professional database. Results revealed several enriched TF binding sites, but SP1 was the only significant TFBS identified across 3 mammalian species. Several putative SP1 matrices were identified and all had very high raw scores, indicating high similarity to consensus SP1 binding sites. We then employed additional approaches to consider whether SP1 or a related TF may be an important regulator of duodenal genes involved in the response to iron-deprivation.

Testing for evolutionary conservation of TFBSs often improves the ability to predict true binding sites from false positives, given that promoters in general show low nucleotide sequence homology across species except in regions that interact with gene regulatory proteins. We therefore determined the locations and specific sequences of the putative SP1 sites from the genes used for the enrichment analysis and compared them across rat, mouse and human. Strikingly, the nucleotide sequences of the SP1 sites were highly conserved and often, multiple putative binding sites existed with the distances between them also conserved across species. This conservation of the SP1 sites was identified in ~40 genes from the large cluster; the actual number of genes could be significantly higher though, as the curators of the Cold Spring Harbor Gene Promoter Database acknowledge that perhaps only 60% of their promoter sequences may be accurate.

SP1 is one of the first transcription factors to be purified, cloned and characterized from mammalian cells [12], and is one of the most potent transcriptional activators identified to date [13,14]. SP1 plays important roles in a wide variety of physiological processes such as cell cycle regulation and apoptosis, hormonal activation, and angiogenesis, among others. SP1 has been shown to interact with GC- rich sites in various gene promoters via three Cys_2_His_2 _zinc-finger motifs [15]. It is now clear that SP1 is a part of a large family of similar zinc-finger transcription factors (also called Krüppel-like factors) that includes 21 identified members in humans, 17 in mouse and 11 in rat [10]. Like other *trans*-acting factors, SP1 has three functional domains: 1) a DNA-binding domain, 2) a nuclear localization signal and 3) a transactivation domain that mediates protein-protein interactions that control transcriptional initiation. Among SP1 family members, the DNA binding domain is highly conserved [16,17], while the transactivation domain varies widely between family members [10]. This high conservation in the DNA binding domains suggests that many of the SP/KLF family members bind highly similar DNA sequences. Our data thus suggests that SP1 or a related TF could be involved in regulating some of the genes in the 228 up-regulated cluster during  iron-deficiency.

Comparison of GeneChip^®^ data from the experimental animal groups considered in this manuscript along with another series of GeneChip^®^ experiments performed in the Belgrade rat model of genetic iron-deficiency (i.e. Dmt1-deficient; not shown), revealed that SP6/KLF14 was induced during both dietary and genetic iron-deficiency. In fact, SP6 was one of < 30 genes that was induced in both models, utilizing stringent statistical analysis approaches. This intriguing observation was confirmed by qRT-PCR analyses that revealed a ~30-fold induction in adult, iron-deficient rats, including experiments performed with 2 different sets of SP6-specific primers. SP6 is a member of the SP/KLF family that was originally identified by a bioinformatics scan of the human genome using the conserved zinc finger DNA binding domains [18]. A subsequent study demonstrated the role of SP6 (also called epiprofin) in dental development in mice [19]. These authors suggested that SP6 binds highly GC-rich regions, similar to other members of the SP/KLF family of DNA binding proteins. Besides this study however, very little is known about SP6 and its target genes. From the current studies we conclude that SP6 is highly induced during iron-deficiency, thus making it a strong candidate for being involved in the genetic response to iron-deficiency.

Further analyses suggested that an SP1-like factor and FOX TFs may function synergistically to regulate some genes during iron-deficiency. The presence of several genes in  the up-regulated cluster with SP1-like and FOX binding sites strengthens this prediction. Like the SP/KLF transcription factors, the FOX family of DNA binding protein has multiple members [20].

## Conclusion

In summary, utilizing a unique GeneChip^®^ dataset and a variety of computational approaches has allowed us to predict that some genes important for the response to iron-deficiency in the rodent intestine are transcriptionally regulated by SP1 or a related TF. This prediction combined with the experimental observation that SP6/KLF14 is strongly induced by iron-deficiency suggests that SP6 could be involved in this genetic response. Further computational analysis allows us to hypothesize that a FOX family DNA binding protein may also be involved in this regulation.

## Methods

### Experimental Animals and GeneChip^®^ Studies

Sprague Dawley rats used for these studies were described in detail previously [3]. Rats at various ages were fed modified AIN-93G rodent diets (Dyets Inc.; Bethlehem, PA), which contained either 198 ppm Fe (DYET# 115135; control diet) or 3 ppm Fe (DYET# 115102; low Fe diet). The diets were identical except for the addition of pure ferric citrate to the control diet. Rats were supplied with food and tap water *ad libitum*. For all studies, only male rats were used and groups of 3–5 animals were considered as one group (n = 1).

GeneChip^®^ experiments were also described in detail previously [2,3]. Briefly, RNA was purified from duodenal and jejunal scrapes and enzymatically converted to cRNA for reaction with Affymetrix gene chips (RAE230A and RAE230B). Experiments at each age were repeated three times with samples derived from separate groups of control and experimental rats. GeneChip^®^ data have been deposited in the GEO repository with accession # GSE1892.

### GeneChip^®^ Data Processing and Analysis

Six experimental groups were studied along with corresponding control groups: duodenum of suckling (8-days-of-age), weanling (21-days-of-age), adolescent (6 weeks-of-age) and adult rats (12- and 36 weeks-of-age), and jejunum of the 12-week-old group only. For data generated from 72 Affymetrix Gene Chips (**2 **chips [RAE230A and RAE230B] × **6 **groups × **2 **conditions [control and iron-deficient] × **3 **repetitions = **72 **chips), we utilized the default *RMA *function included in the "Affy" package of Bioconductor in the *R *statistical computing environment [21]. This default function employs *median polish *for expression summary and quantile normalization for data normalization. We also used *MAS5.0 *"present calls" to filter out probe sets whose expression intensities were close to the background noise across the majority of the samples, before performing the differential gene analysis. We applied the filtering of at least two "present calls" out of 3 replicated samples in either the control or iron-deficient rat groups. This led to a 52.5% – 57.4% data reduction for the comparisons. To detect gene expression values significantly different between groups, we employed *SAM *software [22] by controlling the false discovery rate (FDR) < 0.04 and expression fold changes > 1.5.

### Clustering Analysis

For clustering analysis, we used 1484 probe sets whose expression values were significantly different between the control and experimental groups in at least one postnatal developmental stage of duodenum or jejunum. We built a 1484 × 6 matrix of average fold change between iron-deficiency and control rat groups and used this as input for clustering analysis. Two clustering algorithms were used; K-mean clustering (cluster number set at 3) and Hierarchical clustering, with Euclidean distance as the distance matrix for both algorithms. Based on biological knowledge of the genetic effect of iron-deficiency in gut function [2,3,23], two of the resulting clusters were selected for further analyses; one cluster of up-regulated genes from K-mean clustering contained 228 probe sets, representing ~163 unique genes, and another cluster of up-regulated genes from Hierarchical clustering (called Cluster 7) contained 29 probe sets, representing 25 unique genes.

### Search for Iron-Response Elements (IREs)

For genes in the above two targeted clusters, we obtained the corresponding rat mRNA sequences from GenBank, searching in particular for ones that contained full 3' untranslated regions (UTRs) (as evidenced by the poly A tail). For the predicted rat genes without an identifiable 3' UTR, we used the orthologous mouse genes as replacements. In the case of genes with multiple reference sequences, we compared the mRNA sequences independently for each gene to get the one with the longest 3' UTR (which was more likely to be full length). The resulting 3' UTRs of all genes in each cluster were submitted to UTRscan [24] to identify putative IREs.

### Acquiring Promoter Sequences

We downloaded promoter sequences within 1-kb upstream of the annotated transcription start site (TSS) for each gene in the target clusters for rat, mouse, and human from Cold Spring Harbor Laboratory Mammalian Promoter Database [25]. For genes with multiple mRNA reference sequences, we selected the one with the TSS determined from experimentally identified promoter regions (Database of Transcriptional Start Sites) [26]. In the cases of a reference sequence with alternative promoters, we selected the one defined as the "best" by Xuan et. al. [25]. Out of 163 unique up-regulated genes from K-mean clustering, we were able to obtain promoter sequences for 133, 144, and 159 genes for rat, mouse, and human, respectively. For the 25 unique up-regulated genes from hierarchical clustering, we were able to get promoter sequences for 20 rat, 23 mouse, and 21 human genes. For background sequences, we used randomly selected promoter sequences from Cold Spring Harbor Laboratory within 1-kb upstream of the TSS for 9,201 rat, 9,475 mouse, and 12,367 human unique mRNA RefSeq genes.

### Enrichment Analysis of Transcription Factor Binding Sites

We employed the program CLOVER [9] and 565 vertebrate position weight matrices (PWM) in TRANSFAC (version 9.1) to conduct a search for enriched single TFBSs. Three sets of promoter sequences were used for enrichment analysis of the 163 up-regulated genes from K-mean clustering: 1) 133 rat genes, 2) 144 mouse genes, and 3) 159 human genes. We set the parameters of CLOVER for 1,000 randomizations and a *p*-value threshold of 0.05. For the estimation of *p*-values, we supplied 3 sets of background sequences to the algorithm, which estimates *p*-values for each background set separately (only *p *values from the species corresponding to that for the experimental promoters are reported in the *Results *section). The background sequences included were promoter sequences from rat, mouse, and human (described above).

We employed a 3-step approach to control for false positives. We first selected TFBSs with *p*-values < 0.05 across all 3 sets of background sequences, and then computed the average *p*-values for each TFBS in each set of promoter sequences. Since TRANSFAC contains multiple PWMs, we then applied the method of false discovery rates for multiple test correction to adjust *p*-values as shown in the following formula:

q−value=N∗PcR,
 MathType@MTEF@5@5@+=feaafiart1ev1aaatCvAUfKttLearuWrP9MDH5MBPbIqV92AaeXatLxBI9gBaebbnrfifHhDYfgasaacPC6xNi=xI8qiVKYPFjYdHaVhbbf9v8qqaqFr0xc9vqFj0dXdbba91qpepeI8k8fiI+fsY=rqGqVepae9pg0db9vqaiVgFr0xfr=xfr=xc9adbaqaaeGacaGaaiaabeqaaeqabiWaaaGcbaGaemyCaeNaeyOeI0IaemODayNaemyyaeMaemiBaWMaemyDauNaemyzauMaeyypa0tcfa4aaSaaaeaacqWGobGtcqGHxiIkcqWGqbaudaWgaaqaaiabdogaJbqabaaabaGaemOuaifaaiabcYcaSaaa@3DBE@

where *N *is the number of PWMs from TRANSFAC, and *R *is the ascending rank order of the respective *p*-value at a certain cutoff *P*_*c*_. We selected those TFBSs with *q*-value < 0.1, for the average *p*-values from the 3 sets of promoter sequences. Finally, we selected the enriched TFBSs which intersected among the above selected rat, mouse, and human comparisons.

### Synergistic TFBS analysis

To predict whether any two individual TFs may interact to co-regulate genes, we sought to determine if any combinations of TFBSs were present in the same promoter sequence of the genes in our cluster more often than in a randomly selected group of unrelated gene promoters. We first obtained background probability of TFBS pairs in randomly selected rat, mouse, or human promoter sequences (*PS*) within 1-kb upstream of the TSS according to the following formula:

pf=#genes−with−TFpairs#PS
 MathType@MTEF@5@5@+=feaafiart1ev1aaatCvAUfKttLearuWrP9MDH5MBPbIqV92AaeXatLxBI9gBaebbnrfifHhDYfgasaacPC6xNi=xI8qiVKYPFjYdHaVhbbf9v8qqaqFr0xc9vqFj0dXdbba91qpepeI8k8fiI+fsY=rqGqVepae9pg0db9vqaiVgFr0xfr=xfr=xc9adbaqaaeGacaGaaiaabeqaaeqabiWaaaGcbaGaemiCaa3aaSbaaSqaaiabdAgaMbqabaGccqGH9aqpjuaGdaWcaaqaaiabcocaJiabdEgaNjabdwgaLjabd6gaUjabdwgaLjabdohaZjabgkHiTiabdEha3jabdMgaPjabdsha0jabdIgaOjabgkHiTiabdsfaujabdAeagjabdchaWjabdggaHjabdMgaPjabdkhaYjabdohaZbqaaiabcocaJiabdcfaqjabdofatbaaaaa@4C25@

For this analysis, we utilized 9,201 promoter sequences for rat, 9,475 for mouse, and 12,367 for human, downloaded from Cold Spring Harbor Laboratory. We then computed the number of genes whose promoter sequences contain combinations of two TFBSs in the cluster. Finally, we calculated the probability of observing an equal or larger number of gene promoters in the cluster with both TFBSs than in randomly selected promoter sequences by chance, by summing the binomial distribution probabilities:

P=∑x=nN(Nx)pfx(1−pfx)N−x,
 MathType@MTEF@5@5@+=feaafiart1ev1aaatCvAUfKttLearuWrP9MDH5MBPbIqV92AaeXatLxBI9gBaebbnrfifHhDYfgasaacPC6xNi=xI8qiVKYPFjYdHaVhbbf9v8qqaqFr0xc9vqFj0dXdbba91qpepeI8k8fiI+fsY=rqGqVepae9pg0db9vqaiVgFr0xfr=xfr=xc9adbaqaaeGacaGaaiaabeqaaeqabiWaaaGcbaGaemiuaaLaeyypa0ZaaabCaeaadaqadaqaauaabeqaceaaaeaacqWGobGtaeaacqWG4baEaaaacaGLOaGaayzkaaaaleaacqWG4baEcqGH9aqpcqWGUbGBaeaacqWGobGta0GaeyyeIuoakiabdchaWnaaDaaaleaacqWGMbGzaeaacqWG4baEaaGccqGGOaakcqaIXaqmcqGHsislcqWGWbaCdaqhaaWcbaGaemOzaygabaGaemiEaGhaaOGaeiykaKYaaWbaaSqabeaacqWGobGtcqGHsislcqWG4baEaaGccqGGSaalaaa@4AE6@

where *n *is the number of genes in the cluster with both TFs, and *N *is the total number of genes in the cluster. We selected TFBS pairs with *p* < 0.05 across rat, mouse, and human.

### qRT-PCR

Male Sprague-Dawley rats were acquired at 3-weeks-of-age and fed either a control diet with 198 ppm Fe or an identical diet with 3 ppm Fe. When animals were ~12-weeks-of-age, they were sacrificed by CO_2 _narcosis followed by cervical dislocation. The low Fe animals were obviously iron-deficient as evidenced by pale eyes and extremities, piloerection and pale internal organs. Animals on this dietary regiment also exhibit microcytic, hypochromic iron-deficiency anemia, characterized by decreased hematocrit and hemoglobin levels [2,3]. Mucosal scrapes were taken from the duodenum, consisting of ~20 cm of the small intestine distal to the pyloric sphincter, and snap frozen in liquid nitrogen and subsequently stored at -80°C. Tissue from 2–3 animals in the iron-deficient or control groups was pooled and RNA was purified by Trizol reagent. RNA was quantified by UV spectrophotometry and was enzymatically converted to cDNA using the iScript kit (BioRad; Hercules, CA). cDNA was subsequently used as a template for real-time PCR using SYBR Green mix (BioRad) and primers specific for *SP6*. One primer set was designed based upon the target sequence on the Affymetrix Rat Genome RAE230B array (probe set ID 1381534_at) and one primer set was designed based upon the rat *SP6 *cDNA sequence in the GenBank (Accession # XM_001081357). The primer sequences were as follows: SP6 forward primer 1 5'-TGT-GCT-ACC-AAG-ACA-ACC-TT-3'; SP6 reverse primer 1, 5'-AAG-TGG-GTT-CAC-AGC-AGT-T-3'; SP6 forward primer 2, 5'-GGA-CAT-GTC-ACA-CCA-CTA-CGA-ATC-3'; SP6 reverse primer 2, 5'-ACA-GAG-CTG-CTC-GTC-TCC-GA-3'. *18S *rRNA primers were utilized as constitutive controls. These primer sequences were as follows: 18S forward primer, 5'-TAC-CTG-GTT-GAT-CCT-GCC-A-3'; 18S reverse primer, 5'-TCC-AAG-GAA-GGC-AGC-AGG-C-3'. *18S *levels were very similar between all groups (not shown), indicating that *18S *expression was not affected by iron status of the animals. All primers had no sequence homology with other known genes, as determined by BLAST searches. Preliminary experiments comparing DNAse treated to non-DNAse treated samples showed no differences in *SP6 *amplification, demonstrating that amplicons were not the result of amplification from genomic DNA. This control was especially important as the *SP6 *gene does not contain introns (NCBI GeneID: 363672). PCR amplification parameters were 42 cycles of 58°C annealing for 20 seconds and 72°C extension for one minute. Melt curves were routinely run and single peaks were detected indicating that only one template was being amplified (not shown).

Each RT reaction was analyzed in duplicate for both *18S *and *SP6 *in each experiment. Then, the *18S *average was subtracted from the *SP6 *average to generate the ΔC_t _value. Data were analyzed by routine methods. Briefly, ΔΔC_t _values from each gut segment were calculated from *SP6 *C_t _and *18S *C_t _for the iron-deficient groups vs. the control groups. The ΔΔC_t _was the exponent of 2 for mean fold induction; its standard deviation was the exponent of 2 as an estimate of range. Statistical analyses were done by *t *test.

## Authors' contributions

J.F.C. performed the GeneChip^®^ experiments and the qRT-PCR for SP6 expression, and also performed extensive analysis of computational data as well as drafting the manuscript. Z.H. analyzed GeneChip^®^ data, performed clustering analysis and TF enrichment analyses, and also worked on drafting and editing the manuscript. Both authors read and approved the final manuscript.

## Supplementary Material

Additional file 1 228 Up-Regulated Gene Cluster From the 6 Iron-Deficient Experimental Groups. This table includes the information about the genes in the 228 upregulated cluster and indicates which species promoters were identified from for each gene. Click here for file

Additional file 2 Gene Alignments with Sp1 and Fox and Sp1 binding sites. This file shows sequence alignments of genes in the upregulated gene cluster that have conserved Sp1 and FOX binding sites. Click here for file
