# Supplementary material for: Promoter analysis of intestinal genes induced during iron-deprivation reveals enrichment of conserved SP1-like binding sites
Source: BMC Genomics. 2007 Nov 15;8:420. doi: 10.1186/1471-2164-8-420 (PMC2220005; doi:10.1186/1471-2164-8-420)
Supplement: Additional file 1 — 228 Up-Regulated Gene Cluster From the 6 Iron-Deficient Experimental Groups. This table includes the information about the genes in the 228 upregulated cluster and indicates which species promoters were identified from for each gene. [file 1471-2164-8-420-S1.pdf]

Additional File 1: 228 Up-Regulated Gene Cluster From the 6 Iron-Deficient Experimental Groups

Probe Set ID	Symbol	Gene Name	Promoters
1380577_at	Abcg2	ATP-binding cassette, sub-family G (WHITE), member 2	R, M, H
1372462_at	Acat2	Acetyl-Coenzyme A acetyltransferase 2	R, M, H
1370028_at	Ace	Angiotensin 1 converting enzyme	None
1387791_at	Ace	Angiotensin 1 converting enzyme	None
1393048_at	Adra2a	Adrenergic receptor, alpha 2a	H
1367982_at	Alas1	Aminolevulinic acid synthase 1	None
1387796_at	Alox15	Arachidonate 12-lipoxygenase	R, M, H
1383155_at	Als2cr13	Amyotrophic lateral sclerosis 2 (juvenile) chromosome region, candidate 13 (predicted)	M, H
1384279_at	Als2cr13	Amyotrophic lateral sclerosis 2 (juvenile) chromosome region, candidate 13 (predicted)	M, H
1375815_at	Ank	Progressive ankylosis homolog (mouse)	R, M, H
1385709_x_at	Ank	Progressive ankylosis	R, M, H
1373685_at	Ankrd37	Ankyrin repeat domain 37; Similar to Lrp2bp-pending protein	R, M, H
1386938_at	Anpep	Alanyl (membrane) aminopeptidase	R, M, H
1368981_at	Aqp4	Aquaporin 4	M, H
1372190_at	Aqp4	Aquaporin 4	M, H
1369871_at	Areg	Amphiregulin	R, M, H
1386466_at	Arfgap3	ADP-ribosylation factor GTPase activating protein 3 (Arfgap3)- same chr. Region, mouse	M, H
1368672_at	Arg2	Arginase 2	None
1382802_x_at	Arl2	ADP-ribosylation factor-like 2 (Arl2); same chr. region, mouse; Endogenous retrovirus mRNA- similar	None
1369268_at	Atf3	Activating transcription factor 3	R, M, H
1369342_at	Atp7a	ATPase, Cu++ transporting, alpha polypeptide	R, M, H
1392536_at	Atp7a	ATPase, Cu++ transporting, alpha polypeptide	R, M, H
1378134_at	Atp8b1	ATPase, Class I, type 8B, member 1- similar	R, M, H
1391693_at	Atp8b1	ATPase, Class I, type 8B, member 1 (predicted)	R, M, H
1387184_at	Axin2	Axin2	R, M, H
1390429_at	Axin2	Axin2	R, M, H
1371923_at	Aytl2	Acyltransferase like 2 (predicted)	R, M, H
1375941_at	Baiap2l1	BAI1-associated protein 2-like 1	R, M, H
1388742_at	Bcl2l11	BCL2-like 11 (apoptosis facilitator)	R, M, H
1376640_at	C10orf38	Similar to hypothetical protein (predicted); Astroprincin- same chr. region (human)	R, M, H
1376441_at	C14orf135	Chromosome 14 open reading frame 135; Similar to RIKEN cDNA 1810048J11 (predicted)	R, M, H
1384966_at	C2orf37	C2orf37	H
1385204_at	C2orf37	C2orf37	H
1372237_at	C9orf24	Chromosome 9 open reading frame 24; Testes development-related NYD-SP22 isoform 1- similar (predicted)	H
1390801_at	C9orf71	Chromosome 9 open reading frame 71; Hypothetical protein MGC34760- similar	R, M, H
1389636_at	Camta1	Calmodulin binding transcription activator 1	None
1393452_at	Car9	Carbonic anhydrase 9	None
1391481_at	Cast	Calpastatin	None
1368037_at	Cbr1	Carbonyl reductase 1	R, M, H
1369814_at	Ccl20	Chemokine (C-C motif) ligand 20	R, M, H
1384191_at	Cdgap	Cdc42 GTPase-activating protein	None
1380340_at	Cdkl2	Cyclin-dependent kinase-like 2 (CDC2-related kinase); same chr. region, mouse	None
1398365_at	Cgi-38	Brain specific protein	R, M, H
1375933_at	Cldn2	Claudin 2	R, M, H
1387856_at	Cnn3	Calponin 3, acidic	R, M, H
1391656_at	Cpm	Carboxypeptidase M- 83% similar to mouse	R, M, H
1389659_at	Ctla2b	Cytotoxic T lymphocyte-associated protein 2 beta precursor	None
1368167_at	Ctse	Cathepsin E	R, M, H
1376344_at	Cybrd1	Cytochrome b reductase 1- similar	R, M, H
1377369_at	Cybrd1	Cytochrome b reductase 1 (predicted)	R, M, H
1387020_at	Cyp51	Cytochrome P450, subfamily 51	R, M, H
1368025_at	Ddit4	DNA-damage-inducible transcript 4	R, M, H
1369279_at	Dhrs9	Dehydrogenase/reductase (SDR family) member 9	R, M, H
1378001_at	Dhrs9	Dehydrogenase/reductase (SDR family) member 9- similar; Retinol dehydrogenase isoform 2- homolog	R, M, H
1373291_at	Dlc1; Acot12	Deleted in liver cancer 1 protein homolog; StAR-related lipid transfer protein 12; acyl-CoA thioesterase 12	R, M, H
1390145_at	Dmxl2	Dmx-like 2	R, M, H
1376937_at	Doc1	Downregulated in ovarian cancer 1- similar	M, H
1367966_at	Dpp3	Dipeptidylpeptidase 3	R, M, H
1387414_at	Duox2	Dual oxidase 2	R, M, H
1368146_at	Dusp1	Dual specificity phosphatase 1	R, M, H
1368147_at	Dusp1	Dual specificity phosphatase 1	R, M, H
1374797_at	Eaf1	ELL associated factor 1 (predicted)	R, M, H
1374585_at	Echdc1	Enoyl Coenzyme A hydratase domain containing 1	R, M, H
1388698_at	Ecm1	Extracellular matrix protein 1	R, M, H

Page 2

Probe Set ID	Symbol	Gene Name	Promoters
1387658_at	Eef2k	Eukaryotic elongation factor-2 kinase	None
1372844_at	Efna1	Ephrin A1	R, M, H
1398273_at	Efna1	Ephrin A1	R, M, H
1390763_at	Efna3	Ephrin A3	R, M, H
1368174_at	Egln3	EGL nine homolog 3 (C. elegans); Hypoxia-inducible factor prolyl hydroxylase 3	R, M, H
1368321_at	Egr1	Early growth response 1	R, M, H
1388424_at	Eif3s1	Eukaryotic translation initiation factor 3, subunit 1 alpha (predicted)	M, H
1388666_at	Enc1	Ectodermal-neural cortex 1	R, M, H
1398333_at	Epas1	Endothelial PAS domain protein 1	R, M, H
1389297_at	Ero1l	ERO1-like (S. cerevisiae)	R, M, H
1375908_at	Eva1	Epithelial V-like antigen 1 (predicted)	R, M, H
1385529_at	Fam3b	Family with sequence similarity 3, member B (predicted)	M, H
1381632_at	Fbxl10	F-box and leucine-rich repeat protein 10; same chr. region, mouse	None
1375640_at	Fkbp9	FK506 binding protein 9	R, M, H
1385268_at	Flj10996	Similar to 4633402D15Rik protein	H
1385269_s_at	Flj10996	Similar to 4633402D15Rik protein	H
1376293_at	Flj21865	Endo-beta-N-acetylglucosaminidase- similar	R, M, H
1382235_at	Flj30596	Hypothetical protein FLJ30596, similar	None
1370829_at	Fntb	Farnesyltransferase, CAAX box, beta	R, M, H
1375043_at	Fos	FBJ murine osteosarcoma viral oncogene homolog	M, H
1382729_at	Garnl3	GTPase activating RANGAP domain-like 3 (Garnl3)	M, H
1373386_at	Gjb2	Gap junction membrane channel protein beta 2	None
1372452_at	Gpam	Glycerol-3-phosphate acyltransferase, mitochondrial	R, M, H
1382986_at	Gpam	Glycerol-3-phosphate acyltransferase, mitochondrial	R, M, H
1376828_at	Gprc5a	G protein-coupled receptor, family C, group 5, member A	R, M, H
1374070_at	Gpx2	Glutathione peroxidase 2	R, M, H
1372524_at	Greb1	GREB1 protein	R, M, H
1376076_at	Hig2	Hypoxia induced gene 2- 85% to mouse	M, H
1375852_at	Hmgcr	3-hydroxy-3-methylglutaryl-Coenzyme A reductase	R, M, H
1370080_at	Hmox1	Heme oxygenase (decycling) 1	None
1390480_at	Hrasls5	Similar to H-rev107-like protein 5; HRAS-like suppressor family, member 5	R, M, H
1374558_at	Icoslg	Inducible T-cell co-stimulator ligand; Similar to B7-like protein GL50-B (predicted)	M, H
1368878_at	Idi1	Isopentenyl-diphosphate delta isomerase	None
1388872_at	Idi1	Isopentenyl-diphosphate delta isomerase	None
1367894_at	Insig1	Insulin induced gene	None
1372097_at	Irf8	Interferon consensus sequence binding protein 1	None
1371186_at	Itga6	Intergin alpha 6	R, M, H
1383240_at	Itga6	Integrin, alpha 6	R, M, H
1393558_at	Itga6	Integrin, alpha 6	R, M, H
1383880_at	Itgav	Integrin, alpha V (vitronectin receptor, alpha polypeptide, antigen CD51)	M, H
1382439_at	Itgb6	Integrin, beta 6	R, M, H
1370975_at	Jmjd1a	Jumonji domain containing 1A	R, M, H
1387160_at	Kcne3	Potassium voltage-gated channel, Isk-related subfamily, member 3	R, M, H
1370538_at	Lama3	Laminin, alpha 3	R, M, H
1391022_at	Lamb3	Similar to Lamb3 protein; laminin, beta 3	R, M, H
1379340_at	Lamc2	Similar to laminin gamma 2 chain precursor	R, M, H
1379889_at	Lamc2	Laminin, gamma 2 (predicted)	R, M, H
1376746_at	Ldhd	Lactate dehydrogenase D (predicted)	R, M, H
1382061_at	Ldhd	Lactate dehydrogenase D (predicted)	R, M, H
1387164_at	Lect1	Leukocyte cell derived chemotaxin 1	R, M, H
1376694_at	Lmbr1	Lipocalin-1 interacting membrane receptor- 88% to mouse	R, M, H
1391736_at	Lmbr1	Lipocalin-interacting membrane receptor- similar (predicted); limb region 1 homolog (mouse)-like	R, M, H
1368054_at	Lmna	Lamin A	R, M, H
1368055_a_at	Lmna	Lamin A	R, M, H
1389464_at	Lnx1	Ligand of numb-protein X 1 (predicted); Multi-PDZ-domain-containing protein- similar	R, M, H
1387043_at	Lypd3	GPI-anchored metastasis-associated protein homolog	None
1370948_a_at	Marcks	Similar to Myristoylated alanine-rich C-kinase substrate	None
1392484_at	Moap1	Modulator of apoptosis 1; same chr. region	R, M, H
1375990_a_at	Msl2l1	Male-specific lethal 2-like 1 (Drosophila)- similar	None
1371237_a_at	Mt1a	Metallothionein	R, M, H
1388271_at	Mt2	Metallothionein-2	M, H
1388147_at	Muc3	Mucin 3	M, H
1374001_at	NanogPc	Retrotransposon NANOGPC gene; same chr. region	None
1375230_at	Nat8	N-acetyltransferase 8 (camello like)	None
1392627_x_at	Nat8	N-acetyltransferase 8 (camello like)	None

Probe Set ID	Symbol	Gene Name	Promoters
1374799_at	Ncapd2	Non-SMC condensin I complex, subunit D2	None
1391555_at	Ncoa3	Nuclear receptor coactivator 3	M, H
1392385_at	Ncoa3	Nuclear receptor coactivator 3	M, H
1374650_at	Nedd9	Neural precursor cell expressed, developmentally down-regulated gene 9 (predicted)	R, M, H
1396053_at	Nedd9	Neural precursor cell expressed, developmentally down-regulated gene 9 (predicted)	R, M, H
1370408_at	Nid67	Putative small membrane protein NID67	R, M, H
1384423_at	Nt5c2	5'-nucleotidase, cytosolic II (predicted)	R, M, H
1373412_at	Nt5c3	5'-nucleotidase, cytosolic III (predicted)	R, M, H
1382776_at	Oact1	Similar to O-acyltransferase (membrane bound) domain containing 1 (predicted)	M, H
1378814_at	Osbpl2	Oxysterol binding protein-like 2	R, M, H
1370954_at	P4ha1	Procollagen-proline, 2-oxoglutarate 4-dioxygenase (proline 4-hydroxylase), alpha 1 polypeptide	M, H
1386917_at	Pc	Pyruvate carboxylase	H
1377042_at	Pcgf5	Strongly similar to ring finger protein (C3HC4 type) 159 [Homo sapiens]; polycomb group ring finger 5	M, H
1367671_at	Pcna	Proliferating cell nuclear antigen	R, M, H
1368079_at	Pdk1	Pyruvate dehydrogenase kinase 1	R, M, H
1373272_at	Pepp2	Phosphoinositol 3-phosphate-binding protein 2	R, M, H
1367743_at	Pfkl	Phosphofructokinase, liver, B-type	R, M, H
1387361_s_at	Pgk1	Phosphoglycerate kinase 1	R, M, H
1388318_at	Pgk1	Phosphoglycerate kinase 1	R, M, H
1381576_at	Pik3c2b	Phosphoinositide-3-kinase, class 2, beta polypeptide (predicted)	R, M, H
1383286_at	Plek2	Pleckstrin 2 (predicted)	R, M, H
1379536_at	Plekha5	Similar to phosphoinositol 3-phosphate-binding protein-3; pleckstrin homology domain containing, family A member 5	R, M, H
1370247_a_at	Pmp22	Peripheral myelin protein 22	R, M, H
1370504_a_at	Pmp22	Peripheral myelin protein 22	R, M, H
1381850_at	Ppp1r12a	Protein phosphatase 1, regulatory (inhibitor) subunit 12A	R, M, H
1384262_at	Ppp1r3b	Protein phosphatase 1, regulatory (inhibitor) subunit 3B	R, M, H
1373006_at	Prp2	Proline-rich protein PRP2	R, M, H
1386394_at	Prps1	Ribose-phosphate pyrophosphokinase I -like; phosphoribosyl pyrophosphate synthetase 1- similar	R, M, H
1387085_at	Prps1	Phosphoribosyl pyrophosphate synthetase 1	R, M, H
1390769_at	Prps1	Phosphoribosyl pyrophosphate synthetase 1- similar	R, M, H
1393241_at	Prss32	Protease, serine, 32 (Prss32)	None
1392953_at	Ptpla	Protein tyrosine phosphatase-like (proline instead of catalytic arginine), member a	None
1398768_at	Rbbp7	Retinoblastoma binding protein 7	R, M, H
1371583_at	Rbm3	RNA binding motif (RNP1, RRM) protein 3	R, M, H
1373488_at	Rbms1	Similar to RNA binding motif, single stranded interacting protein 1 isoform c	M, H
1375870_a_at	Rbms1	RNA binding motif, single stranded interacting protein 1 (predicted)	M, H
1387240_at	Rdh7	Retinol dehydrogenase 7	R, M, H
1377751_at	Reps2	RALBP1 associated Eps domain containing protein 2	R, M, H
1369958_at	Rhob	RhoB gene	R, M, H
1372197_at	Rictor	Pianissimo- similar; rapamycin-insensitive companion of mTOR	R, M, H
1373427_at	Rragd	Ras-related GTP binding D (predicted)	R, M, H
1382835_at	Rragd	Unkown EST clone; Ras-related GTP binding D (Rragd)	R, M, H
1370428_x_at	RT1-Aw2	RT1 class Ib, locus Aw2	None
1367668_a_at	Scd2	Stearoyl-Coenzyme A desaturase 2	R, M, H
1367849_at	Sdc1	Syndecan 1	R, M, H
1376062_at	Sdc1	Syndecan 1	R, M, H
1376973_at	Sdcbp2	Syndecan binding protein (syntenin) 2	R, M, H
1380283_at	Sdccag33	Serologically defined colon cancer antigen 33 (predicted)	R, M, H
1383573_at	Sdccag33	Serologically defined colon cancer antigen 33 (predicted)	R, M, H
1387046_at	Slb	Selective LIM binding factor, rat homolog	M, H
1367877_at	Slc11a2	Solute carrier family 11 (proton-coupled divalent metal ion transporters), member 2	R, M, H
1388059_a_at	Slc11a2	Solute carrier family 11 (proton-coupled divalent metal ion transporters), member 2	R, M, H
1367853_at	Slc12a2	Solute carrier family 12, member 2	R, M, H
1376969_at	Slc23a2	Solute carrier family 23 (nucleobase transporters), member 2	None
1380167_at	Slc23a2	Solute carrier family 23 (nucleobase transporters), member 2	None
1383913_at	Slc30A10	Zinc transporter 8; Znt8	R, M, H
1383971_at	Slc30A10	Zinc transporter 8; Znt8	R, M, H
1368778_at	Slc6a6	Solute carrier family 6 (neurotransmitter transporter, taurine), member 6	R, M, H
1374531_at	Slc6a6	Sodium-dependent taurine transporter	R, M, H
1380265_at	Snapc1	Small nuclear RNA activating complex, polypeptide 1 (predicted)	R, M, H
1376649_at	Snf1lk2	Salt-inducible kinase 2	M, H
1382291_at	Sox9	SRY sex determining region Y-box 9 protein- similar	R, M, H
1385978_at	Sox9	SRY-box containing gene 9	R, M, H
1381534_at	Sp6	Trans-acting transcription factor 6; Epiprofin	R, M, H
1370884_at	Spr	Sepiapterin reductase	R, M, H

Probe Set ID	Symbol	Gene Name	Promoters
1374864_at	Spry2	Sprouty-2- similar	None
1387017_at	Sqle	Squalene epoxidase	R, M, H
1372510_at	Srxn1	Sulfiredoxin 1 homolog (S. cerevisiae)	R, M, H
1373164_at	Stk17b	Serine/threonine kinase 17b (apoptosis-inducing)	R, M, H
1387502_at	Stk17b	Serine/threonine kinase 17b (apoptosis-inducing)	R, M, H
1393956_at	Stk17b	Serine/threonine kinase 17b (apoptosis-inducing)	R, M, H
1384220_at	Tbcc	Tubulin-specific chaperone c (predicted)	R, M, H
1377156_at	Tcf7l2	TCF7L2 protein- similar	R, M, H
1379815_at	Tcf7l2	Transcription factor 7-like 2, T-cell specific, HMG-box	R, M, H
1394497_at	Tcf7l2	Transcription factor 7-like 2 (HMG box transcription factor 4) (T-cell-specific transcription factor 4) (TCF-4)	R, M, H
1373808_at	Tead1	TEA domain family member 1 (SV40 transcriptional enhancer factor)- similar	R, M, H
1389287_at	Tead1	TEA domain family member 1 (SV40 transcriptional enhancer factor)	R, M, H
1389409_at	Tes	Transcribed locus, strongly similar to NP_035700.2 testis derived transcript [Mus musculus]	None
1371113_a_at	Tfrc	Transferrin receptor	R, M, H
1388750_at	Tfrc	Transferrin receptor	R, M, H
1373421_at	Tgif	TG interacting factor (predicted)	R, M, H
1367712_at	Timp1	Tissue inhibitor of metalloproteinase 1	R, M, H
1390832_at	Tmcc3	Transmembrane and coiled coil domains 3- similar	R, M, H
1393822_at	Tmcc3	Transmembrane and coiled coil domains 3- similar	R, M, H
1377234_at	Trim27	Tripartite motif protein 27 (predicted)	M, H
1371737_at	Trim27; Rfp	Tripartite motif protein 27; Ret finger protein	R, M, H
1376284_at	Trim66	Tripartite motif-containing 66- similar; Similar to transcriptional intermediary factor 1 delta	None
1374133_at	Trit1	tRNA isopentenyltransferase 1 (predicted)	R, M, H
1385641_at	Tube1	Similar to tubulin, epsilon 1; epsilon-tubulin	R, M, H
1390259_at	Ube2d1	Similar to ubiquitin-conjugating enzyme E2D 1, UBC4/5 homolog; ubiquitin-conjugating enzyme E2D 1	M, H
1374684_at	Xist	Inactive X specific transcripts	None
1375535_at	Xist	X specific transcripts	None
1386785_a_at	Xist	Inactive X specific transcripts	None
1386721_at	Zfp503	Zinc-finger protein NOLZ1	R, M, H
1378688_at	Znf213	Zinc finger protein 213 (predicted)	None
1371394_x_at		Endogenous retroviral sequence, 5' and 3' LTR	None
1376304_at		Transcribed locus, strongly similar to XP_236949.2 similar to hypothetical protein BC006605 [Rattus norvegicus]	None
1379660_at		Unkown EST clone	None
1381556_at		Transcribed locus, moderately similar to XP_358375.2 cDNA sequence BC013672 [Mus musculus]	H
