# Supplementary material for: Promoter analysis of intestinal genes induced during iron-deprivation reveals enrichment of conserved SP1-like binding sites
Source: BMC Genomics. 2007 Nov 15;8:420. doi: 10.1186/1471-2164-8-420 (PMC2220005; doi:10.1186/1471-2164-8-420)
Supplement: Additional file 2 — Gene Alignments with Sp1 and Fox and Sp1 binding sites. This file shows sequence alignments of genes in the upregulated gene cluster that have conserved Sp1 and FOX binding sites. [file 1471-2164-8-420-S2.pdf]

Additional File 2: Gene Alignments with Sp1 and Fox and Sp1 binding sites

Blue Font- Sp1 binding sites, **Green font**- FOX binding sites; **Orange numbers**- distances between putative sites. H- human, M- mouse, R- rat. Promoter sequences go from 0-1000, with 1000 being closest to the transcription start site.

Abcg2
M--839-ctccgccctc-848-(31)-879-ccccgcccac-888-(47)-935-cggggcggcg-944
R--889-ctccgccctc-898-(31)-929-ccccgcccac-938-(47)-985-cggggcggcg-994

Ankrd37
H--857-agggggcgggcc-869
R--877-agggggcggagc-889

Aqp4
M--468-cccccacccc-477
R--641-cccccacccc-650

Atf3
H----098-aaaaaaaaaaaat-110-(407)-517-ccccgccccc-526-(150)-766-gagggcgggc-685-----------------------------795-cccctcctcc-804
M----195-taaaaataaaaac-207-(222)-429-ccccgccccc-438-(298)-736-gagggcgggc-745-(100)-845-ccccgccccc-854----867-cccctcctct-876
R----175-taaaaacaaaaat-187-(386)-573-ccccaccccc-582-(146)-728-gagggcgggc-737-(100)-837-ccccgccctc-846

Atp7a
H---------------------------844-ggagggaggggt-856-(23)-879-gggggtgggx-888-(11)-899-agagggaggagtt-911
M--79-ttatgttttatt-91-(796)-894-ggagggcggggc-905-(07)-912-gtgggctggg-921-(09)-930-cgagggaggagct-942
R--25-ttatgttttatt-36-(790)-826-ggcgggcggggc-837-(07)-845-gtgggctggg-854-(24)-878-cgagggaggagct-891

Axin2
H--755-gggggaggag-764-(136)-900-gggggcgggc-909-(6)-915-cggggcgggg-924
M--792-gggggaggag-801-(136)-937-gggggccggg-946-(2)-948-cggggcgggg-957

Bcl2l11
H--727-cggggtgggg-736---881-ggggcggag-890----924-gggggcgggg-933
M--701-cggggagggg-710-706-aggggcgggt-715-(71)-786-gtgggcggcg-795-(84)-879-gggggtgggg-888-(35)-923-gggggcggag-932----951-gggggcgggg-960
R---------------------731-aggggcgggt-740-(71)-811-gtgggcggcg-820-(84)-904-gggggtggga-913-(35)-948-gggggcggag-957

Ccl20
M--782-ctcctcccct-791-(3ol)--788-ccctgccctc-797-(5ol)-792-gccctcccca-801-(4ol)-797-ccccaccccc-806-(8ol)-798-cccaccccc-807
R--798-ctcctcccct-807-(3ol)--804-ccctgccctc-813-(5ol)-808-gccctcccca-817-(4ol)-813-ccccaacccc-822-(8ol)-814-cccaaccccc-823

Cgi-38
H--835-aggggcggga-844-868-gagggcgggg-877-(5)-882-gtgggcgggg-891
M--241-ccacacccac-250--463-ggggtgggg-472--614-aggggcggga-623-862-tagggcgggg-871-(5)-876-gtgggaggag-885-(40)-925-gggggtggga-934
R--366-ccacacccag-375--521-gggggtggag-530---------------------913-tagggcgggg-922-(5)-927-gtgggaggag-936-(40)-975-gggggtggga-984

Cnn3
H---750-ggggcggggc-759---------------------------------793-xxgggcggggx-902
M--192-ttaaaaaacaaac-204-(8ol)-196-aaaacaaaccaaa-208-(38)-246-aaaacaaataaac-258-(486)-744-ggggcggggc-753-(23)-776-gggaggaggggcg-788-(19)-807-tgagggcggggcc-819
R--220-taaaaaaacaaac-232-(8ol)-224-aaaacaaacaaaa-236-(38)-274-aaaacaaacgaac-286-(451)-737-ggggcggggc-746-(23)-769-gggaggaggggcg-781-(19)-800-tgagggcggggcc-812

Cybrd1
H------------------------------860-cggggtgggggcc-872-(67)-940-atccctcccc-949
M--635-taaggaaacaagg-647-(163)-810-aaggggtgggacc-882-(60)-882-gcccctcccc-891
R--635-taaggaaacaagg-647-(155)-802-aaggggtgggacc-814-(60)-874-gcccctcccc-883

Cyp51
H--594-gggggcggtg-603-(329)-932-tcccgccccca-941
M--593-gggggcggtg-602-(324)-926-ccccgccccg-935
R--574-gggggcggtg-583-(350)-933-ccccgccccg-942

Ddit4
M--698-cccctccccc-707-(109)-816-ggggtgtgg-825-(55)-880-gggggcgggg-889
R--698-cccctccccc-707-(109)-816-ggggtgtgg-825-(55)-880-gggggcgggg-889

Additional File 2- page 2

Efna3
H--912-ggggcggggc-921-(17)-938-ggagggcggaggg-950
M--891-ggggcggggc-900-(17)-917-gaagggcggaggg-929
R--630-ggggcggggg-639------809-agcgggcggcgcg-821

Egr1
H--239-ccccgcctct-248-(440)-688-gagggagggg-697
M--266-ccccgcctct-275-(418)-693-ggaggagggg-702-(12)-714-tggggcgggg-723
R--277-ccccgcctct-286-(407)-693-ggaggagggg-702-(12)-714-tggggcgggg-723

Ero1l
H--565-cggggcggcg-574--575-cggggcgggc-584---732-gggggcgggg-741
M--697-cggggcgggg-706--702-cggggcgggc-711-(40)-752-cccgaccccg-761-(134)-895-ggaggcggga-904-(13)--918-agggcggag-926-(03)-929-cggggcgggg-938
R--756-cggggcgggg-765--771-cggggcgggc-780-(40)-820-ccccgacccc-829-(120)-950-ggaggcggga-959-(13)--973-agggcggag-981-(10)-991-cggggcgggg-1000

Fntb
H---881-gggggcggcagc-893-(30)-923-ggcgggaggagac-935
M--256-aaatatttatgt-267-(128)-395-gaatatttgttga-407-(455)-862-gggggcggcagt-874-(32)-906-ggtgggaggagag-918
R--305-aaatatttatgt-316-(128)-444-gaatatttgttga-456-(425)-881-gggggcggcagt-893-(32)-925-ggtgggaggagag-937

Rictor
H--879-ggggcgggg-887-(12)-899-gggggcggg-907-(9)-917-cggggagggg-926
M--902-ggggcgggg-910-(25)-935-gggggcggg-943-(9)-953-cggggagggg-962

Gjb2
M--779-gggggtgggg-788-(11)-799-tggggtgggg-808-(82)-890-gggggtggcg-899-(52)-951-agaggcgggg-960
R--773-gggggtgggg-782-(11)-793-tggggtgggg-802-(81)-883-gggggtggcg-892-(52)-944-agaggcgggg-953

Gpx2
H--669-tccctccctcc-681-(105)-786-agggtggggc-800
M----------------------------787-ggggtggggc-796
R--782-tccctcccttc-794-(102)-896-ggggtggggc-905

Hmox1
H--875-ggggcgggct-884
R--910-ggggcggggt-919

Jmjd1a
H-486-ccctccccc-494--------------------804-gggggagggg-813-815-aagggaggag-824-(29)-853-ccccgccctc-862-(91)-953-gggggcgggt-962
M-501-ccctccccc-509-526-ccccgcccct-535-821-gggggaaggg-830-826-aagggaggag-835-(29)-864-ccccgccctc-873-(90)-963-gggggcgggt-972
R-------------------006-ccccgcccct-015-705-gggggcgggg-714---987-gggggtgggg-996

Mt1a
H----------------------------914-ggggcgggcg--940
M--604-tgggcggagc-613-(199)-812-aggggcggtccc-824-(33)-857-actccgcccg-866
R--582-tgggcggagc-594-(215)-809-aggggcggtcc-821-(33)-854-actccgcccg-863

Nt5c3
M--398-ccccacccac-407-(271)-678-gtgggtggcg-687-(199)-886-tcccgccccc-895-(52)-947-ccccgcccct-956-(9)-965-ctgggcgggg-974
R--313-ccccacctac-322-(271)-593-gtgggtggcg-602-(189)-791-tcccgcccct-800-(53)-853-ccccgcccct-862-(9)-871-ctgggcggag-880

Pcna
H---------------------832-gccccgccct-841---------------869-ggggcgggcc-881
M--834-gcccctcgcc-843-864-gccccgcctt-873-(43; 843-886)-886-ggggcgggcc-895
R--890-gcccctcgcc-899--------------(43)----------------942-ggggcgggcc-951

Pgk1
H--744-ctccgcccct-753-------------(178)-------------931-aggggcggtg-940
M--380-tactttttattat-392-(6)-398-gggggagggg-407-(152)-559-aggggaggcg-568-(152)-720-ctcctcccct-729-(206)-935-aggggcgggc-944-(03)-947-aggggcgggg-956
R--423-tactttttattat-435-(2)-437-aggggcgggg-446-(153)-599-gggggcggcg-608-(142)-750-ctcctcccct-759-(173)-932-aggggcgggc-941-(14)-955-aggggcgggg-964

Ph4a1
H--841-cgggtggggg-850
M--714-cgggcggggg-723-(123)-846-gtgggtgggagg-858
R--642-ggggtggggg-654-(112)-766-gtgggtgggagg-778

Additional File 2- page 3

Pmp22
```
H--894-gaccagcccc-903
R--991-gaccagcccc-1000
```

Rhob
```
H--578-cccctccccc-587-(160)-747-gaaggagggg-756-855-ccctgccccg-864-861-cccgccc-867
M--577-cccctccccc-586-(161)-747-gaaggcgggg-756-819-cccagccccg-828
R--492-cccctccccc-501---------------------------------------------973-cccgccc-979
```

Sdc1
```
H--872-gggggtgggg-881-(20)-912-ggggcgggg-920------------------------------------------------967-gggggtgggg-976
M--874-gggggtgggg-883-(09)-892-ggggcgggg-900-908-gggggcgggg-917-(5)-922-aggggtggag-931-(28)-959-gggggtgggc-968
R--827-gggggtgggg-836------------------------861-gagggcggag-870-(5)-875-aggggaggag-884-(28)-912-gggggtgggc-921
```

Slc11a2
```
H----------------------------197-aattttttttttttt-209-(8ol)-201-ttttttttttttta-213-----------------------------------------878-tgggcggagcc-892
M--150-ttctgtttgctt-161-(09)-170-atttttttttttttt-181-(6ol)-175-ttttttttttttta-187-232-ttaccaaataatg-244-338-ggggcgggg-346-852-tgggcggagcc-866
R--369-ttctgtttgtct-380-(18)-398-atttttttttttttx-408-----------------------------455-ttaccaaataatg-467-637-ggggcgggc-646
```

Slc6a6
```
H----------------------------------------------------------------------------939-ccccgccccg-948-(2)-950-ccccgccccc-959
M--309-accctcccag-318-(255)-573-gggggaggag-582-(329)-911-gtgggcgtgg-920-(14)-934-cccctcccta-943-(3)-946-ccccgcccag-955
R--240-accctcccag-249-(241)-490-gggggaggag-499-(332)-831-gtgggcgtgg-840-(14)-854-ccccgcccta-863-(3)-866-ccccgcccag-875
```

Spr
```
H---------------------894-ccccgccc-901
M--826-gtgggagtgg-835-913-ccccgccc-920-(138)-973-ctccgccccg-982
R--790-gtgggagtgg-799---------(144)----------943-ctccgccccg-952
```

Tbcc
```
H---------------------667-gggggagggg-676-(139)-815-ccccacccat-824-(19)-843-tcccaccccc-852-(13)-865-ccccgcccca-874-(4)-878-ccccgccccc-887
M--081-tcccgcccca-090-625-gggggagggg-634-(152)-786-ccccacccat-795-(19)-814-ccccaccccc-823-(14)-837-ccccgccctc-846-(3)-849-tcccgcccct-858
R--260-tcccgcccca-269-628-gggggagggg-637-(148)-785-ccccacccat-794-(19)-813-ccccaccccc-822-(14)-836-ccccgccctc-845-(3)-848-ccccgcccct-857
```

Tfr
```
H----------------------------------------------------------951-cgggggcggggcc-963
M--357-ttttatttgctat-369-(387)-756-cgggggaggggtg-768-(185)-952-gggggtggggcc-964
R--181-ttttatttgctat-193-(381)-574-cgggggaggggtg-586-(184)-771-cgggggcggggcc-783
```

Tgif
```
H-----------------------------------------------------------------------------------------------------949-gcgggcgggg-958
M--386-gaatagacattt-397-(158)-555-ccccacccct-564-(337)-901-gggggagggg-910-(18)-928-gcgggcgggg-937-(17)-954-gggggcggag-963
R--021-gaatagacattt-032-(171)-203-ccccacccct-212-(333)-545-gggggagggg-554-(16)-570-gagggcgggg-579-(17)-596-gggggcggag-605
```

Timp1
```
H-----------------------------------------------------------------------------------------------------834-agaggaggag-843-(20)-863-gcccgcccct-872
M--89-agggtgtgg-100-(11)-112-ggggaaggg-120-(416)-536-cactatgtatttg-548-(260)-808-gggggaaggg-817-------838-agaggaggga-847-(23)-870-gcccgcccct-879
R--90-agggtgtgg-101-(11)-113-ggggaaggg-121-(437)-558-cactatgtatttg-570-(275)-845-gggggaaggg-854-------------------------------906-gcccgcccct-915
```

Trim27
```
H-----------------------------------------------------918-agccccgcccccg-930
M--650-actgggaggggc-662-(232)-894-ttcccgcccc-903-(28)-931-ggccccgcccccg-943
R--453-actgggaggggc-465-(231)-696-ttcccgcccc-705-(25)-730-ggccccgcccccg-742
```
